# User Perceptions of eHealth and mHealth Services Promoting Physical Activity and Healthy Diets: Systematic Review

**DOI:** 10.2196/34278

**Published:** 2022-06-28

**Authors:** Julia Bergevi, Susanne Andermo, Yohannes Woldamanuel, Unn-Britt Johansson, Maria Hagströmer, Jenny Rossen

**Affiliations:** 1 Department of Health Promoting Science Sophiahemmet University Stockholm Sweden; 2 Department of Neurobiology, Care Sciences, and Society Karolinska Institutet Stockholm Sweden; 3 Department of Public Health Sciences Karolinska Institutet Stockholm Sweden; 4 Department of Clinical Sciences and Education Karolinska Institutet Stockholm Sweden; 5 Academic Primary Health Care Center Stockholm Sweden

**Keywords:** acceptability, behavior change, engagement, health technology, noncommunicable diseases, usability, user feedback, qualitative studies, physical activity, healthy diet

## Abstract

**Background:**

Physical activity and a diet that follows general recommendations can help to prevent noncommunicable diseases. However, most adults do not meet current recommended guidelines, and support for behavior change needs to be strengthened. There is growing evidence that shows the benefits of eHealth and mobile health (mHealth) services in promoting healthy habits; however, their long-term effectiveness is uncertain because of nonadherence.

**Objective:**

We aimed to explore users’ perceptions of acceptability, engagement, and usability of eHealth and mHealth services that promote physical activity, healthy diets, or both in the primary or secondary prevention of noncommunicable diseases.

**Methods:**

We conducted a systematic review with a narrative synthesis. We performed the literature search in PubMed, PsycINFO, and CINAHL electronic databases in February 2021 and July 2021. The search was limited to papers published in English between 2016 and 2021. Papers on qualitative and mixed method studies that encompassed eHealth and mHealth services for adults with a focus on physical activity, healthy diet, or both in the primary or secondary prevention of noncommunicable diseases were included. Three authors screened the studies independently, and 2 of the authors separately performed thematic analysis of qualitative data.

**Results:**

With an initial finding of 6308 articles and the removal of 427 duplicates, 23 articles were deemed eligible for inclusion in the review. Based on users’ preferences, an overarching theme—eHealth and mHealth services provide value but need to be tailored to individual needs—and 5 subthemes—interactive and integrated; varying and multifunctional; easy, pedagogic, and attractive; individualized and customizable; and reliable—emerged.

**Conclusions:**

New evidence on the optimization of digital services that promote physical activity and healthy diets has been synthesized. The findings represent users’ perceptions of acceptability, engagement, and usability of eHealth and mHealth services and show that services should be personalized, dynamic, easily manageable, and reliable. These findings can help improve adherence to digital health-promoting services.

## Introduction

Noncommunicable diseases, such as type 2 diabetes, cardiovascular diseases, and certain types cancer (colon, breast, prostate), are the leading causes of impaired quality of life and premature death worldwide, responsible for 71% of all deaths globally [1]. In Europe, where 60% of incidences are associated with unhealthy lifestyles (such as poor diet and physical inactivity) [2], close to 800,000 EU citizens die yearly because of noncommunicable diseases. The noncommunicable disease epidemic continues to grow and is expected to cause 75% of all global deaths by 2030 [3]. World Health Organization guidelines on physical activity and sedentary behavior suggest that adults should perform at least 150 to 300 minutes of moderate-intensity aerobic exercise or 75 to 150 minutes of vigorous-intensity aerobic exercise per week [4]. If adults were more physically active, 4 to 5 million global deaths yearly could be prevented [4]. Yet, only 1 in 4 adults meet the global recommendations for physical activity [5]. There is also growing evidence that a healthy diet plays an important role in preventing noncommunicable diseases [6]. Dietary recommendations may vary between nations but originate from global guidelines [6] that suggest that adults should eat all macronutrients in balance with the energy expenditure; consume a limited amount of saturated fats, trans fats, sugars, and salt; and consume more fruits, vegetables, and whole grains.

eHealth has been defined as “the use of emerging information and communications technology to improve or enable health and health care [7].” A subsegment of eHealth is mobile health (mHealth), which has been defined as “medical and public health practice supported by mobile devices, such as mobile phones, patient monitoring devices, personal digital assistants, and other wireless devices [8].” eHealth has the potential to support behavior change, and thus, improve health. For instance, several studies that have investigated the effects of digital lifestyle interventions reported short-term positive effects in disease-specific clinical outcomes [9-11], physical activity levels [12,13], and dietary patterns [12,14]. There is also evidence that physical activity interventions delivered using technology are 12% more effective in increasing physical activity levels than those that are not delivered using technology [15]. However, 75% of people who download smartphone health apps stop using the apps within a short time [16]. There is a need to identify factors which influence engagement with and adherence to health-promoting technology [10,11,17,18].

Previous reviews of qualitative studies have captured users’ perceptions and beliefs about mHealth apps [19] or analyzed different behavior change techniques and persuasive system designs in concern of users’ motivation and maintenance in eHealth tools [20]. However, to our knowledge there is no summarized evidence on users’ perceptions of factors that may affect the acceptability, engagement, and usability in eHealth and mHealth services that focus exclusively on physical activity, diet, and lifestyle-related diseases in the primary and secondary prevention of noncommunicable diseases. Filling this gap is vital to capitalize on the promising prospects of health technology. Therefore, this systematic review explores users’ perceptions of acceptability, engagement, and usability of eHealth and mHealth services that promote physical activity, healthy diets, or both in the primary or secondary prevention of noncommunicable diseases.

## Methods

### Overview

In this systematic review, qualitative studies were summarized using a narrative synthesis [21]. The process followed the PRISMA (Preferred Reporting Items for Systematic Reviews and Meta-analyses) framework [22] (checklist [23] in Multimedia Appendix 1). Only studies with ethical approval were included to avoid encouraging eHealth interventions in which study participants may have harmed their physical or mental health. The review was registered on July 25, 2021 (PROSPERO International Prospective Register of Systematic Reviews; CRD42021261844).

### Search Strategy

**Figure 1:**
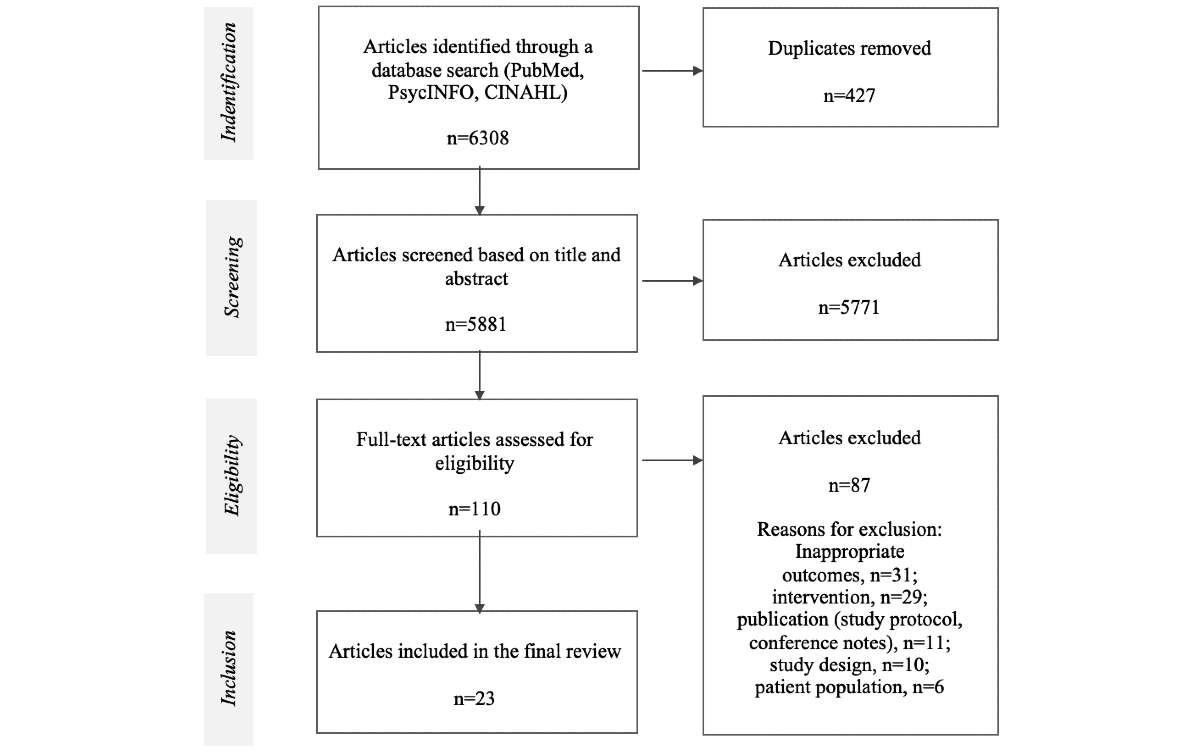
Search strategy.

Assisted by 2 university librarians, we searched PubMed, PsycINFO, and CINAHL electronic databases in February 2021; we updated the search results in July 2021 (Figure 1). The search terms were (“acceptability” OR “engagement” OR “usability” OR) AND (“digital service” OR “eHealth” OR “mHealth”) AND (“behavior change” OR “physical activity” OR “diet”). A full overview of the search terms is listed in Multimedia Appendix 2. The search was limited to papers published in English between 2016 and 2021, given that the rapidly progressing nature of health-promoting technology [24] likely lowered the relevance of older publications (ie, outdated technology). All identified studies were imported to review management software (Covidence systematic review software, Veritas Health Innovation) that automatically removed duplicates. Three authors (JB, YW, JR) independently screened titles and abstracts to determine whether papers would be included in the second screening phase. Any disagreements were discussed. In the second screening phase, the full texts were independently screened by 2 authors (JB, JR) to determine the final selection of papers.

### Selection Criteria

The selection criteria (Table 1) were based on the PEO (Population, Exposure, Outcome) framework [25]. We included papers describing qualitative or mixed methods studies that presented qualitative data on acceptability, engagement, and the usability of digital behavior change services for the promotion of physical activity, healthy diets, or both, consistent with current World Health Organization guidelines [4,6]. We did not include papers that focused on interventions that targeted sedentary behaviors only.

**Table 1:** Selection criteria based on the PEO (Population, Exposure, Outcome) framework.

Criterion	Inclusion	Exclusion
Study type	Ethical approval, full text available, English language, all geographical locations, published between 2016 and 2021	No ethical approval, full text not available, non-English language, published before 2016
Study design	Original qualitative study, original or secondary analysis of a mixed methods study including a qualitative method	Systematic review, meta-analysis, study protocol, efficacy or effect study evaluating effect only
Population	Healthy adults (≥18 years) and adults with noncommunicable diseases, including overweight or obesity, type 2 diabetes, cardiovascular diseases and relevant cancer types (colon, breast, prostate)	Children and adolescents (≤18 years), pregnant women, clinical populations (eg, communicable diseases and severe diseases)
Exposure	Web-based platform or mobile app promoting lifestyle-related behavior changes on an individual level focusing on physical activity, healthy diets, or both in the primary or secondary prevention of noncommunicable diseases	Focus not explicitly on physical activity, healthy diets, or both (alcohol, tobacco, sleep, sedentary behavior, mental health, medical adherence), physical activity or diet not comparable to WHO^a^ guidelines, behavior changes at group level (eg, group-oriented activities in workplace settings), content adapted to a specific clinical population (eg, cancer, cardiovascular diseases patients) and thus not suitable for general adults, platform or app including dietary recording, calorie counting, exergames, social networking, short message service, digital counseling, wearables only (ie, no multicomponent platforms or apps)
Outcome	Qualitative data on acceptability, engagement, and usability	Quantitative data on acceptability, engagement, and usability; qualitative data on a user’s perceived effect or general experience in participating in a study; qualitative data on an individual’s general preferences of eHealth technology (ie, no platform or app yet designed); health care professionals’ perceptions; qualitative data only evaluating one feature of a platform or app

^a^WHO: World Health Organization.

### Data Extraction and Analysis

Data extraction was performed by JB and reviewed by JR in accordance with Guidance on the Conduct of Narrative Synthesis in Systematic Reviews [21]. The procedure included three steps: (1) tabulation to provide detailed information of all studies in a common table (Multimedia Appendix 3), including reference details, study design, population, exposure, outcome, and quality; (2) clustering to organize the findings into groups relevant to the research aim (participants’ subjective opinion clustered as facilitators, barriers, and suggested improvements); and (3) translation to explore similarities and differences between the studies using thematic analysis (ie, identify the most relevant and important themes and concepts across the studies in an inductive manner, hence, without predefined themes to guide the analysis) [21]. The thematic synthesis included 3 steps, described by Thomas et al [26] as “coding of text line-by-line; the development of ‘descriptive themes’; and the generation of ‘analytical themes.’”

### Quality Appraisal

Quality appraisal was performed in accordance with the process described in The Swedish National Agency for Medical and Social Evaluation method book [27]. Quality criteria (authors’ affiliated departments, study design, study theory, recruitment, data collection, data analysis methodology, relevance to the study aim, coherence, sample size, and results) were assessed as high, medium, or low.

## Results

### Overview

A total of 6308 papers were identified; 427 duplicates were removed, 5881 titles and abstracts were screened for eligibility, and 110 full-text articles were read. The final sample comprised 23 papers (Figure 1).

### Study Characteristics

Study characteristics are outlined in Multimedia Appendix 3. Papers were published between 2016 and 2021, with the majority published after 2018 (n=18). The studies were undertaken in the United Kingdom [28-33], Italy [34], Belgium [35,36], Finland [37,38], Portugal [39], Sweden [40], Germany [41], the United States [42-46], Australia [47-49], and Canada [50]. Of the included studies, 11 were qualitative studies [32,34,35,37,40,42-44,46,47,50], and 12 were mixed methods studies [28-31,33,36,38,39,41,45,48,49]. Most studies included participants of both sexes [28-41,43,44,46,47,49,50], except for 1 study [48] with men only, and 2 studies [42,45] with women only. One study did not report sex distribution [39]. In total, sex was reported for 417 women, 309 men, and 1 nonbinary individual. In the studies with both men and women, most often, women were overrepresented. In the 23 studies, 769 participants between 18 and 75 years (mean age range 34-62 years) were included. Ten studies included healthy adults [28,31,34-36,38-40,46,49,50], and 13 included adults with obesity or who were overweight [29,30,33,37,42,45,47,48], adults with type 2 diabetes [43], or adult cancer survivors [29,32,44].

More than half of the studies focused on apps [28,29,31,32,34,35,38,40,42,44,45] or websites [36,41] promoting physical activity, while the rest involved diet-promoting apps [30,33,49], or diet- and physical activity–promoting apps [39,43,46-48,50] or websites [37]. The most commonly applied behavior change techniques according to behavior change technique taxonomy [51] were (presented in descending order) feedback and monitoring (category 2) [28-31,33,35-42,44-48], goals and planning (category 1) [29-31,35,36,38,40,44,47,48,50], social support (category 3) [28,31,37,44,47,48], and rewards and threats (category 10) [29,31,33,38,44,48]. The services were tested for up to a 1-year period, with the majority (n=18) being tested for ≤4 months. One study [44] did not report the intervention period’s duration. Of the 23 studies, 20 studies used semistructured interviews [29-41,44-50], 2 studies used think-aloud interviews [34,39], 5 studies used focus group discussions [30,33,42,43,45], and 3 studies used web-based questionnaires [39,41,49].

### Study Quality

In all studies, a theoretical framework was used to support the purpose of the study; papers included information on authors with relevant professions, recruitment methods, data collection and analysis, and results: 10 studies were appraised as high quality [32,34,35,37,40,42,46,47,49,50], 11 studies were appraised as medium quality [29-31,36,38,39,41,43-45,48], and 2 studies were appraised as low quality [28,33] (Multimedia Appendix 3).

### Findings

#### Overview

An overarching theme and 5 subthemes emerged from the thematic analysis reflecting users’ perceived acceptability, engagement, and usability of eHealth and mHealth services promoting physical activity and healthy diets. The overarching theme showed that eHealth and mHealth services provide value but need to be tailored to create compelling services that offer long-term user value. The subthemes indicated that users prefer services to be (1) interactive and integrated; (2) varying and multifunctional; (3) easy, pedagogic, and attractive; (4) individualized and customizable; and (5) reliable.

#### Provide Value but Need to Be Tailored

Users recognize that eHealth and mHealth services can support behavior change but that more effective services are needed to meet individual needs, provide long-term user value and keep engagement over time.

#### Interactive and Integrated

Users stressed the importance of an interactive service, enhanced with a dynamic bidirectional communication path between the service and the user. Some users described dynamic communication as a desire to make services more human and less robotic, for instance, to make services operate as an automatic coach or to integrate services with a physical coach [35]. Several users expressed disappointment when the service was not sufficiently interactive or did not provide sufficient support [29,32,34-36,38,39,41,46,47]. More interactive guidance was expressly requested when including goal setting, action planning, and coping planning [35,36,46,47]. The request for more interactive guidance was exemplified by 2 users who stated desire for and satisfaction with interactive guidance when discussing physical activity–promoting apps. One user said,

Something that gradually guide you toward your goals, step-by-step, perhaps also suggesting what kind of physical activity to do and providing advice.

34

The other user remarked,

It provides suggestions about how much activity to do per week, how to increase it, etc. That what I liked a lot.

34

Integration with health experts, external health devices, and support services to increase user engagement and usability was feedback commonly expressed by users. Some users wanted to connect with personal trainers, health coaches, and clinicians to receive information, recommendations, and feedback [32,34,37,48]. One study suggested inviting expert moderators to create more productive discussions when social networking [37]. Some studies reported that the integration of other device apps (eg, calendar, alarm, and external health apps) as suggestions for improvement [39,40,49]. Some users found it comfortable to track physical activity by phone [42,46], whereas others preferred the integration of an app with a wearable [29,31,34,35,47]. In addition, users appreciated automatic syncing with external apps or wearables that monitor multiple variables (eg, steps, distance, calories, heart rate) were integrated [35,40-42,47]. Some users proposed services that enable meal planning and food purchasing by integrating people’s shopping lists with a web-based grocery service [49]. Other suggestions for integration included the ability to synchronize app content with family members and friends (eg, sharing goals and grocery lists), connect to sponsors that donate rewards when goals are achieved, obtain community resources and location-specific recommendations to facilitate physical activity, and arrange meetup-style events to gain support from and connect with peers online [29,42-44,47,49].

#### Varying and Multifunctional

Variety was another frequently cited theme of importance. One user stated,

If it always stays the same I think I will not use it for long and will consequently delete it

39

The significance of variety applied both to the content and to the included behavior change techniques of the service. Users preferred variety or novelty over repetition for motivational, inspirational, and educational content [33,34,39-41,44,49]. One user said,

...it was the same exact wording in the message every single time, so it almost seemed like robotic.

44

Apps with several behavior change techniques were appreciated. The behavior change techniques most appreciated by users were social networking [28-31,34-36,38-40,42-45,48], self-monitoring [28-31,33,34,37,38,40,43,47,48,50], push notifications [28-31, 34-37, 40, 44, 47], progress tracking [29,31,34-36,40,44,46], goal setting [34,40-42,44,47,50], and gamification (ie, gamified challenges and rewards) [31,33,35,37,44,47,48]. Users also appreciated the ability to track several health parameters in the same app (eg, energy expenditure, heart rate, weight loss, physical activity level, diet, water intake) [34,40,44,47,50]. One user commented,

I would have liked an app that includes a wide variety of health measurements. Now there are apps for movement and apps for eating, but if you got them all in one app I would use it a lot more. If the app included other health components, I could have set goals that were more attractive to me.

40

Yet, one study [38] found that services with too many options may be a hindrance for older people (≥63 years), which could potentially affect user acceptability and usability negatively. Another study [49] reported that it might be problematic to include to many features, as this could make the service difficult to navigate.

#### Easy, Pedagogic, and Attractive

Users recognized the value of a straightforward service with good flow and a menu that can be easily navigated [31,32,34-36,39,45-47,49,50]. At the same time, they disliked cognitively demanding or time-consuming services [30,34,43,48]. One user said,

I mean part of the reason why the step app worked so well was that you literally turn it on it does everything. There isn’t really a lot I need to do to interact with it further.

46

Another user noted,

Y’know...it’s a nice, simple app. You don’t need to be that literate.

32

Users often reported manual data entry as an obstacle because it was time-consuming [34,35,37,43,48], especially in tracking physical activity or diet using a diary [37,41,43,48]. Users preferred easily performed exercises that did not require additional equipment [32,41]. In one study that used photos of meals for dietary self-monitoring, users found the method to be inappropriate in social settings [33]. Another study applied self-monitoring of food choices using “Happy-scores [39].” Users liked its easy and educational way to monitor and reflect on lifestyle habits. One user said,

We saw when we said we ate “bad” foods (fried food and such), and we lowered our score, it was...we thought “right, I shouldn’t have eaten that” or “I should have eaten a healthier food.” The fact that we have a score and we see the effect of that score in our behaviour ends up motivating us to have a better score.

39

Visualization of goals and clinical parameters using easy-to-read graphs was either requested or appreciated as a way to track progress in a larger context [30,32,37,40,43,44,50]. In addition, many users wanted to be provided with a manual or initial tutorial to learn about the service or new tools. They also desired technical support [29,31,41,46,47,50]. Users valued the attractiveness of an app if the content and tone of the service are not discouraging or associated with illness and disease. Overall, users preferred services to be encouraging, fun, and positive [29,37-40,44,45,49]. One user said,

Although [another sport app] it’s just an app, but it says something like “now you’ve missed your training session,” it makes me feel somehow bad. So probably you should pay attention to that, how the feedback is.

38

What was perceived as an attractive layout varied widely among the users. While some preferred a clean design [31,35,36,46,50], others favored more color [35-37,48]. One study [42] stressed the importance of using a layout that was not too child-like (eg, excluding smiley faces) as it decrease the service’s relatability. In 2 studies [47,50], changeable layout themes were offered, which the users appreciated. For external physical activity trackers, the users valued small, light, and waterproof devices [41,44].

#### Individualized and Customizable

Several studies [32,35-41,44-46,49,50] reported individualized content as a facilitator or suggestion for improvement when interacting with eHealth and mHealth apps. For example, users valued content tailored to personal motives and goals, current health status, fitness level, motivation level, season, weather conditions, and profile set-ups (such as sex, age, and personal interests) [32,34,36-38,40,41,50]. However, one study [38] reported concerns stereotyping based on interests or activities and emphasized the importance of modifiable individual set-ups. In one study [49], users suggested that recipes should be adapted to the family constellation (eg, modified portion sizes and meal suggestions appropriate to young children). For physical activity–promoting apps, some users noted the importance of offering relevant and challenging exercises [50]. Moreover, addressing users by their names was suggested (eg, when sending push notifications) [45]. Several studies [29,34,35,38,40,45] reported that users like to gain a sense of control of the service by customizing behavior change techniques to personal needs, preferences, and schedules. One user expressed discontent when the push notifications were not tailored to the person’s schedule:

The amount of time is not much, but sometimes it is...because you get the notification at 8 o’clock, that didn’t fit my working schedule. If I start with an early shift, I get up at 5 o’clock in the morning, at 6.30 o’clock I’m already at work...and then I actually have to think about my app during coffee break...And those things didn’t always go so well...

35

In addition, there were mixed opinions on certain elements. For instance, users did not agree with push notifications, social networking, and gamification: some appreciated or requested them [32,34,37,38,40], while others found them inappropriate or annoying [32,34,37,38,40,45]. Some users wanted to adjust push notifications to personal goals, frequency, and time [30,45]. Two studies also emphasized the importance of customizing the content to a user’s self-identity (ie, sex, age, body size, and fitness level) when, for example, sharing activity tips using video clips and internet instructors [32,35]. One user said,

And of course, umm, on both of them [J&J and Gorilla Workout]...the videos, err, show the sort of slim, fit young, ultra-fit, young men doing it. You think, “Gosh, I...I haven’t looked like that for about 40 years.

32

#### Reliable

A reliable service, with proven personal safety and trustworthiness, was expressed as essential. Some users complained about sharing personal data and wanted confidentiality ensured before sharing private and sensitive data [32,43]. A service that originated from a trustful source (such as recognized authorities or health care professionals) and provided evidence-based content in line with public recommendations, was perceived as being more reliable [29,37,46,49,50]. User perception of the reliability of services decreased when excessive advertisements, when parts of the content were unavailable if not paid for, and regular system updates were part of the service [34,35,37,40,44,46]. When tracking the physical activity level by phone or wearable, users reported that it was important for the tracker to be convenient and technical accurate in distinguishing different activities (eg, walking, running, biking) [32,34,40]. Technical issues were generally perceived as impediments, with users expressing the need for apps to be technically stable, easily manageable, and effective. Finally, apps should not drain the battery, mobile data usage, or phone memory [32,44,49,50].

## Discussion

### Principal Results

In this systematic review, we explored adults' perceptions of the acceptability, engagement, and usability of eHealth and mHealth services that focus on physical activity, healthy diets, or both in the primary and secondary prevention of noncommunicable diseases. The results showed that users value eHealth and mHealth services, but considerations need to be taken account to maintain engagement. Users preferred services to be (1) interactive and integrated; (2) varying and multifunctional; (3) easy, pedagogic, and attractive; (4) individualized and customizable; and (5) reliable. By taking these findings into account, we believe that adherence to eHealth and mHealth services could be significantly improved.

### Comparison With Previous Research

Users underlined the need for variation. This user view was supported by Dennison et al [52], who reasoned that new and updated content increases mHealth app users’ motivation and engagement. Users also valued a service that is composed of several behavior change techniques. There was some disagreement about the effectiveness of behavior change techniques and about the number of behavior change techniques that should be employed. A meta-analysis [53] reported that intervention effectiveness increased when more behavior change techniques were included. In contrast, Kelders [54] underscored the importance of matching user and intervention characteristics rather than applying several behavior change techniques. Our findings indicate that users preferred individualized services. Users also valued a straightforward and easy-to-use service, which is aligned with the *less-is-more* strategy for effective human–computer interactions [55]. A service offering multiple behavior change techniques tailored to users’ preferences and activities can enhance user engagement. Broekhuizen et al [56] confirmed that tailoring to an individual’s needs was beneficial in digital health behavior change interventions. On the other hand, tunneling the content and basing it on presumed stereotypical activities should be avoided as presumed assessments may mislead the tailoring process. Analytical and artificial intelligence–based methodologies that use input from the app or captured by external devices, could improve user individualization without increasing user burden [57]. Our findings also show that users valued an interactive service enhanced with bidirectional communication and indicative support, especially when the behavior change techniques were used for goals and planning (category 1 [51]). Evidence supports this finding, showing that it is crucial with indicative support to set realistic and achievable goals when minding motivation and engagement [58]. Some evidence stated the importance of applying well-established behavior change techniques when designing health-promoting technology, with suggestions to include self-monitoring and goal setting as support for physical activity and dietary behavior changes [59-61]. It is well evidenced that self-monitoring and goal setting appear to enhance the behavior change process and increase the intervention effect [59-61]. This review shows self-monitoring of several health parameters, goal setting, social networking, gamification, and push notifications were valued as behavior change techniques. Social networking, gamification, and push notifications were appreciated to gain support and enhance motivation, although some users found these behavior change techniques to be inappropriate or even annoying when not carefully adjusted to personal schedules, motives, and interests. There were conflicting opinions about social networking and gamification. For instance, some studies reported that social networking and gamification favored usability (ie, the efficacy and satisfaction of the service) [62,63], whereas others felt it was not essential for long-term behavior changes [18,64]. However, this difference in findings reflects users’ individual preferences, which suggests that there is a need to offer a customizable and flexible device to provide a personalized and dynamic service that follows the varying attitudes, values, and schedules of users. Some users expressed privacy concerns when sharing personal data, which could be an issue when tailoring is used. Individualization and anonymity have been discussed as a problem in eHealth elsewhere [38,65]. Finally, our results suggest that time efficiency may be another crucial factor that is particularly challenging in monitoring dietary habits. This view was supported by Peng et al [65], who reported that ease of use and time efficiency was significant for long-term engagement to mHealth apps.

### Strengths and Limitations

Our results are based on newly published studies, which is a strength given the rapidly progression of eHealth and mHealth. Another strength is that we included studies with healthy adults and adults with a medical history and from a wide age range. Thus, the results can be generalized as users' perceptions may vary with age and the purpose of the service being used. We also included studies with participants of both sexes and from several countries. However, a slightly larger number of women (417 women compared with 309 men) were included in this review, limiting the generalizability to male populations. Studies were from diverse high-income countries strengthening international generalization. Yet, few studies were from low- and middle-income countries which restricts generalization to low- and middle-income countries. Most of the studies included in this review had recruited participants interested in using health-promoting services. This recruitment bias could limit the generalizability of our results because such individuals are likely to have a level of motivation that is higher than that of the general population. Also, we included studies in which individuals used the digital service for free, which may affect the expectations and perceptions of the service compared with the real world, where consumers pay for services. Most studies had an intervention period of only 4 months, which is a limitation because users’ perceptions are likely to change; however, one with a 12-month intervention period did not report any deviating results. All studies were included regardless of assessed quality; however, none of the studies assessed with low quality added anything new or distinctive to the results. Also, this review only included qualitative studies; quantitatively measured aspects were not considered. Researcher bias is a potential limitation in analyzing qualitative data. However, this limitation is less of an issue, because in our review, 2 authors independently analyzed and discussed the findings.

### Conclusion

Our findings from the synthesis of studies on the optimization of digital services to promote physical activity and healthy diets represent users’ perceptions of acceptability, engagement, and usability and show that eHealth and mHealth services provide value but need to be tailored to make them personalized, dynamic, easily manageable, and reliable. These findings can be useful in improving the user value when receiving support by digital services for behavior change to promote healthy lifestyles and increase adherence to eHealth services.

## Appendix

Multimedia Appendix 1 PRISMA checklist.

Multimedia Appendix 2 Search terms.

Multimedia Appendix 3 Description of studies included in the review.

## Abbreviations

mHealth: mobile health
