# Supplementary material for: User Perceptions of eHealth and mHealth Services Promoting Physical Activity and Healthy Diets: Systematic Review
Source: JMIR Hum Factors. 2022 Jun 28;9(2):e34278. doi: 10.2196/34278 (PMC9277535; doi:10.2196/34278)
Supplement: Multimedia Appendix 2 [file humanfactors_v9i2e34278_app2.docx]

**Multimedia Appendix 2.** Search terms.

| **Users’ perceptions:** |
| --- |
| Patient/user acceptance  Consumer behavior  Acceptability  Usability  Engagement  Maintenance Adherence Compliance Patient/user/client perspective Patient/user/client experience Patient/user/client satisfaction Patient/user/client attitudes Patient/user/client participation |
| **Health technology:** |
| Telemedicine Telehealth  Telenursing Digital service Digital intervention Electronic health mHealth eHealth  App(s)  Mobile health  Mobile applications  Smartphone |
| **Exposure:** |
| Health behavior Health promotion Healthy lifestyle Health knowledge/attitudes/practice Lifestyle change Behavior change  Motivation Self-regulation/control Sedentary behavior  Physical activity  Exercise Diet |
