# Supplementary material for: User Perceptions of eHealth and mHealth Services Promoting Physical Activity and Healthy Diets: Systematic Review
Source: JMIR Hum Factors. 2022 Jun 28;9(2):e34278. doi: 10.2196/34278 (PMC9277535; doi:10.2196/34278)
Supplement: Multimedia Appendix 3 [file humanfactors_v9i2e34278_app3.docx]

**Multimedia Appendix 3.** Description of the 23 articles including study design, population (health status, age, sex, sample size (n)), exposure (description of the digital platform/app and qualitative methodology to explore acceptability, engagement and usability), outcome (participants’ perceptions clustered as ‘facilitators,’ ‘barriers’ and ‘suggested improvements’) and quality appraisal.

| Reference | Study design | Population | Exposure | Outcome | Quality |
| --- | --- | --- | --- | --- | --- |
| Alturki, et al. (48), Australia, 2019 | Qualitative study | Adults with obesity (18-64 years). n=10 (5 males, 5 females). | Semi-structured interviews after 2 weeks of use of the weight loss, diet- and PA^1^ promoting app “Akser Waznk” including educational elements, personalized tips, goal setting, meal planning, self-monitoring, gamification, push notifications, social networking and augmented reality (AR) to scan fitness equipment and foods, which provide information and recommendations on PA^1^ and nutrition. | Facilitators:   - Colorful design with selectable themes - Easily navigated layout - Self-monitoring of weight loss, PA^1^, diet and water intake - Social gamification features (common step goals) - Gamification rewards - Easy-to-use AR   Suggested improvements:   - Add meal-time reminders - Add tutorials on how to use the app - Connect the app to an external wearable - Collaborate with sponsors that donate rewards in achieving a goal | High |
| Baretta, et al. (35), Italy, 2019 | Qualitative study | General adults not meeting the PA^1^ recommendations (mean age 39.8 years). n=20 (11 males, 9 females). | Think-aloud and semi-structured interviews during first exposure and after 2 weeks of using one of three top-ranked, publicly available PA^1^ promoting apps, including behavior change techniques. | Facilitators:   - Simple and easy to use and thus not cognitively demanding or time-consuming - Self-regulation features (self-monitoring, feedback, goal setting) to monitor behaviors, progress over time and gaining a sense of control - Step-by-step action planning - Accurate activity tracker with related variables (calories, heart rate) that distinguish different activities - Content tailored to personal motives - Social incentives for empathic support and health challenges with online peers - Push notifications (reminders, suggestions)   Barriers:   - Manual data entry - Inappropriate reminders - Advertisements - Social networking - Push notifications   Suggested improvements:   - Connect the app with health coaches or personal trainers | High |
| Cesar, et al. (43), USA, 2019 | Qualitative study | Overweight or obese adult women (mean age 62.1 years). n=16. | Semi-structured focus group interviews after 20 days of using the PA^1^ tracker “Fitbit” and the PA^1^ promoting app “Step It Up,” including educational elements, self-monitoring and motivational push notifications. | Suggested improvements:   - Add location-specific recommendations for outdoor training - Connect the app to community resources (suitable and safe training facilitates) to overcome PA^1^ barriers and exercise/walking groups for social support - Show PA^1^ data in the app and not in external wearables - Increase the relatability (less child-like layout, more relatable avatars, i.e. heavier women) - Add more educational content (nutrition, stretching, sleep) and goal setting | High |
| Degroote, et al. (36), Belgium, 2020 | Qualitative study | General adults not meeting the PA^1^ recommendations (mean age 39 years). n=20 (7 male, 13 females). | Semi-structured interviews after 2-weeks of using the PA1 promoting app “MyDayPlan” include action planning, coping planning, self-monitoring and reflection. | Facilitators:   - Easy to use (straightforward, good flow) - Simple and clear layout - Action- and coping planning - Daily goals - Progress tracking - Daily reflection - Time-efficient - Individualized content   Barriers:   - Manual data entry (time-consuming) - Technical issues - Formulating concrete and achievable actions, - Identifying barriers/solutions - Content not personally relevant   Suggested improvements:   - More attractive layout - Add individualized challenges and game elements (e.g., rewards) - Provide guidance and support for action- and coping planning - Minimize manual data entry - Add push notifications and social networking with online peers and family/friends - Connect the app to an external wearable - Individualized content (e.g., personalized goals that are reasonably challenging) | High |
| Du, et al. (44), USA, 2020 | Qualitative study | Adults with type 2 diabetes and obesity (mean age 59.4 years). n=10 (2 males, 8 females). | Semi-structured group discussions after 6 weeks and 6 months using the diet and PA^1^ promoting app “LoseIt!” including self-monitoring of diet, exercise and weight. | Facilitators:   - Visualization of calorie input/expenditure - App integrated with family/friends - Feeling comfortable sharing personal data   Barriers:   - Technical issues - Lack of knowledge/experience in technology - Time-consuming elements (e.g., logging foods) | Medium |
| Forster, et al. (29), UK, 2018 | Mixed-method study | General adults not meeting the PA^1^ recommendations (mean age 47 years). n=9 (2 males, 7 females). | Semi-structured group discussions after 4-months of using the PA-promoting app “Haptivity” where users post photographs when exercising to capture positive emotions, social networking and push notifications. | Facilitators:   - Sharing pictures and dialoguing with online peers - Activity reminders   Suggested improvements:   - Add PA^1^ tracking and calorie counting | Low |
| Gilson, et al. (49), Australia, 2017 | Mixed-method study | Male truck drivers with obesity not meeting the PA^1^ and nutrition recommendations (mean age 47.5 years). n=17. | Semi-structured interviews after 20-weeks of using the activity tracker and diet- and PA^1^-promoting app “Jawbone UP” including self-monitoring, goal setting, social networking and gamification. | Facilitators:   - Social networking - Self-monitoring of PA^1^ and diet - Gamified challenges and rewards.   Barriers:   - Time-consuming (especially diet monitoring) - No support from health care professionals. | Medium |
| Groarke, et al. (30), UK, 2021 | Mixed-method study | Adult cancer survivors with overweight or obesity (38-68 years). n=13 (2 males, 11 females). | Semi-structured interviews after 8-weeks of using the activity tracker “Fitbit” and a PA^1^ promoting app, including self-monitoring, goal setting, gamification and push notifications. | Facilitators:   - Fitbit - Encouraging push notifications - Goal setting - Progress tracking - Trustful source (researchers and health care professionals)   Suggested improvements:   - Avoid stressful notifications - Add educational elements in nutrition - Include dietary and goal setting reminders - Offer support in how to use the app - Deliver notifications on a fixed schedule (same time/day each week) - Provide opportunities to interact and get support from online peers. | Medium |
| Joseph, et al. (46), USA, 2020 | Mixed-method study | African American women with obesity not meeting the PA^1^ recommendations (24-49 years). n=12. | Focus groups or semi-structured interviews after 1-month use of the PA^1^ promoting app “Smart Walk” including self-monitoring, educational texts/videos, multimedia activity materials, a discussion board with weekly topics for reflection and social networking, push notifications. | Facilitators:   - Clear - Fun - Informative   Suggested improvements:   - Send real-time push notifications if online peers share posts on discussion boards - Do not send push notifications as text messages - Detailed profile setting to create a stronger sense of community (name, picture/image, city, brief biography) - Add a discussion board with free dialogs not related to the weekly topic - Make social networking optional - Make messages more individualized by addressing name and allow tailoring (frequency, time) | Medium |
| Karppinen, et al. (38), Finland, 2016 | Qualitative study | Overweight or obese adults (mean age 47.1 years). n=43 (21 males, 22 females). | Semi-structured interviews after 12-months of use of the digital lifestyle program “Onnikka” including self-monitoring (weight graph, food-, exercise- and mood diary), weekly content on different themes, individualized content (information, exercises, tips), push notifications, social networking (discussion forum) and credibility support (external sources). | Facilitators:   - Self-monitoring (especially the weight graph noticing small weight losses) - Individualized content - Reminders - Weekly themes - Trustworthy and credible sources by recognized authorities and content in line with public recommendations - No commercials   Barriers:   - Food diary (time-consuming) - Mood diary and social support (unattractive or uncomfortable) - Plain layout - Content not encouraging enough - Reminders.   Suggested improvements:   - Elaborate social networking: For example, inviting an outside moderator to create lively and meaningful discussions - Add rewards after exercising or eating healthy. | High |
| Lieffers, et al. (51), Canada, 2018 | Qualitative study | General adults (18-70 years). n=26 (3 males, 23 females). | Semi-structured interviews after 1 week of using the diet- and PA^1^ tracking app and website “eaTracker” including self-monitoring and goal setting. | Facilitators:   - Calorie, nutrient, portion size and PA^1^ tracking - Goal setting - Simple layout - Selectable background picture - Review of data from previous days - Trustful source (Dietitians of Canada) - Available as app and website with integrated data - More detailed educational content on the website - PA^1^ and dietary elements separated   Barriers:   - Complex search feature (too detailed, not relevant items/activities) - Limited food database without the ability to customize nutritional variables - Different layout on app and website - No progress graphs or recipes in the app - Content not personalized to goals, personal preferences, fitness level or health status - Technical issues. | High |
| Lindqvist, et al. (41), Sweden, 2020 | Qualitative study | General adults (mean age 51 years). n=17 (4 males, 13 females). | Semi-structured interviews after 3 months using the PA^1^ and active transportation promoting app “TravelVU” including self-monitoring, goal setting and push notifications. | Facilitators:   - Self-monitoring - Weekly goal setting (especially when relevant and within reach) - Daily encouraging reminders - Graphical statistics on activities - Semiautomated activity registration   Barriers:   - Repeating reminders - Unreliable activity tracker   Suggested improvements:   - Send reminders about the weekly goal at the beginning of the week - Offer social networking to share progress and compete with online peers - Make daily reminders optional and more varying - Minimize the technical workload - Individualize the content to specific needs and circumstances (highlight/hide common/uncommon activities, send individualized and season-specific messages) - Display statistics more appealing and interactive - Track calories - Enable connection to other health apps - Make goal setting more adaptable (frequency, time) | High |
| Mauch, et al. (50), Australia, 2021 | Mixed-method study | Healthy and working single and partnered parents (mean age 33.6 years). Questionnaire n=62 (sex distribution not reported), interviews n=36 (1 male, 35 females). | Questionnaire and semi-structured interviews after 4 weeks of using two of five selected commercially available apps promoting healthy diets, including meal planning, recipe storage, shopping lists, food preparation skills, family organizer and barcode scanning. | Facilitators:   - Inspiring and varying recipes - Barcode scanning supporting healthy food choices - Efficient meal planners - Automated generation of shopping lists - Easy to use, i.e. intuitive and self-explanatory requiring low user input - Technically accessible, convenient and effective - Personalized content (e.g., recipes and portion size modified to the family constellation) - The app's purpose is clearly demonstrated - Trustworthy - Visually attractive.   Barriers:   - Features and content not adjusted to the family constellation - Barcode screening not available during online shopping - Time demanding - App trying to serve too many purposes - Low-content apps   Suggested improvements:   - Tailor recipes to the family constellation (e.g., age of children) - Provide nutritional information from national guidelines - Enable modification of automated shopping lists and completion from planning to purchasing by integrating generated shopping lists with online shopping - Sync between devices so that several family members can contribute to food provision-related tasks | High |
| Miropolsky, et al. (45), USA, 2020 | Qualitative study | Adult cancer survivors not meeting the PA^1^ guidelines (20-39 years). n=13 (4 males, 8 females, 1 non-binary). | Semi-structured interviews after using the activity tracker and PA^1^ promoting app “Fitbit” including self-monitoring, goal setting, gamification, push notifications and social networking using a private Facebook group that includes app users and one invited family member or friend. | Facilitators:   - Wearable easy to use (small, light, waterproof) - Calorie tracking - Gamified rewards - Motivational messages with a positive tone - Educational messages (e.g., benefits of PA^1^) - Goal setting - Social networking (following online peer’s goals and activities, compete with and plan activities using the private social media group, sharing the experience and giving/receiving support from individuals with a similar medical history)   Barriers:   - Regular manual updates - Short battery life of wearables - Repetitive and impersonal messages - Unrealistic and unachievable goals   Suggested improvements:   - Elaborate the social network (e.g., by sharing images using Snapchat or Instagram) - Arrange competitions/challenges and meetup-style activities - Make social networking available on the app - Track more variables (e.g., heart rate) - Present weekly activates to view PA^1^ patterns in a larger context - Make messages more personal and varying | Medium |
| Mummah, et al. (31), UK, 2016 | Mixed-method study | Adults with obesity (mean age 42.6 years). n=14 (3 males, 11 females) | Semi-structured interviews were conducted after interacting with two prototype versions of the vegetable promoting app “Vegethon” including self-monitoring only or self-monitoring and habit challenges using goal setting, daily inspiration and push notifications. | Facilitators:   - Self-monitoring - Simple and easy to follow   Suggested improvements:   - Track type of consumed vegetables - Make graphs easier to read if including habit challenges - Add more inspirational and educational elements (practical tips, benefits of a healthy diet) - Personalized and adjustable reminders (frequency, time) - Social networking (arrange competitions with online peers). | Medium |
| Nurmi, et al. (39), Finland, 2020 | Mixed-method study | General adults (25-63 years). n=12 (4 males, 8 females). | Instant think-aloud and semi-structured interviews when or after interacting with the PA^1^ promoting app “Precious” including autonomy support by motivational interviewing, goal setting, action planning, self-monitoring, gamification and push notifications. | Facilitators:   - Self-monitoring - Personalized content and feedback tailored to profile set-ups (sex, age, interests, goals) - Suggested and motivational outcome goals   Barriers:   - Too many elements and options, especially for older users   Suggested improvements:   - Simplify the motivational interview (fewer answer options) - Make profile set-ups changeable and do not tunnel the content to stereotype activities - Avoid discouraging feedback - Provide activity tips | Medium |
| Paul, et al. (32), UK, 2017 | Mixed-method study | Healthy older adults (mean age 71.1 years). n=15 (8 males, 7 females). | Focus group discussions after 6 weeks using the PA^1^ promoting app “STARFISH” including self-monitoring, goal setting, gamification/social networking (fishes swimming in a common sea as users’ activity level). | Facilitators:   - Simple layout - Real-time self-monitoring - Step count history - Gamification/social networking   Barriers:   - Technical issues (low technology experience) - Small text size - PA^1^ tracking by phone   Suggested improvements:   - Provide an app manual - Track more activities than walking (e.g., cycling, wearable). | Medium |
| Poppe, et al. (37), Belgium, 2018 | Mixed-method study | General adults (mean age 46.65 years). n=20 (10 males, 10 females). | Semi-structured interviews after 5 weeks of use of the PA^1^ promoting web-based platform “MyPlan 2.0” where users choose “increase PA^1^” or “decrease SB” as main target behavior, further including action planning, goal setting, push notifications, coping planning and self-monitoring. | Facilitators:   - User-friendly layout (clear and straightforward) - Personalized feedback - Action planning - Barrier identification and problem solving - Goal reviewer - Time-efficient   Suggested improvements:   - More colorful design - More varying content - Tailor the content to current PA^1^ and motivational level - Focus either on PA^1^ or SB (not both) - Provide more guidance on action planning and barrier identification - Add more activity tips - Enable to share success stories with online peers | Medium |
| Riberio, et al. (40), Portugal, 2017 | Mixed-method study | General adults (18-35 years). Questionnaire n=32, interview n=10. | Online questionnaire and semi-structured interviews after 4 weeks of using the healthy lifestyle promoting app “Happy” to prevent cancer, including self-monitoring and push notifications. | Facilitators:   - Easily accessible when available on your smartphone - Layout and content with a positive tone hence not associated with disease - Personalized notifications - The easy and educational way in monitoring lifestyle habits (e.g., healthy foods, physical activities) using “Happy-scores”   Barriers:   - Content is not interactive enough and varying   Suggested improvements:   - Include social networking (arrange exercise/diet groups) - Integrate the app with other apps (e.g. calendar/alarm/fitness apps) - Add motivational push notifications | Medium |
| Roberts, et al. (33), UK, 2019 | Qualitative study | Adult cancer survivors (mean age 60 years). n=32 (22 males, 10 females). | Semi-structured interviews after 2 weeks of using two out of four publicly available PA^1^ promoting apps, including behavior change techniques (1 week per app). | Facilitators:   - Easy exercises (no equipment needed, no cost, not having to attend a specific exercise facility) - Exercise demonstrations by video - Individualized content tailored to goals, motivational level and physical condition - Accurate activity tracker - Trustworthy content (evidence-based) - Push notifications (feedback on behavior, reminders) - Self-monitoring (especially step counting; safe, accessible, achievable hence enjoyable) - Goal setting - Easily navigated layout - Visually interactive (low level of literacy required)   Barriers:   - Unsupervised exercise (worries of being unsafe) - Technical issues (impact on battery, mobile data usage, phone memory) - Data security when sharing personal data - Inappropriate reminders due to time, context and tone - Video instructor’s not matching self-identity (sex, age, body size, fitness level) - Gamification   Suggested improvements:   - Connect app to health care practice to receive information, recommendations and feedback from health care professionals | High |
| Vaghefi, et al. (47), USA, 2019 | Qualitative study | General adults (18-51 years). n=17 (5 males, 12 females). | Semi-structured interviews after 2 weeks of using a publicly available app promoting a healthy lifestyle. | Facilitators:   - Clean and simple design - Easy to navigate - Navigation instructions as an introduction - Self-adjustable (frequency, time) and simple push notifications to presenting progress overviews - Automatic data entry using passive sensing - Convenient data entry with comprehensive databases when automatic data entry is not possible - Flexible goal setting (alternative scales and measurement tools) - Progress tracking - Trustworthy and accurate information - Individualized content.   Barriers:   - Overwhelming advertisements - Extern wearables - Cost-wisely unavailable content - Time-consuming   Suggested recommendations:   - Provide information and actionable recommendations with clear action steps to improve current health conditions and achieve goals | High |
| Whitelock, et al. (34), UK, 2020 | Mixed-method study | Adults with overweight or obesity (mean age 41.7 years). n=38 (7 males, 31 females). | Semi-structured interviews after 8 weeks of using an attentive eating app, including dietary self-monitoring by photos or text, educational push notifications, encouraging audio clips and gamification. | Facilitators:   - Rewards - Meal monitoring by text - Attractive self-monitoring photo gallery   Barriers:   - Inappropriate to photograph in social settings - Social desirability bias - Forgetting to track meals - Recurring audio clips | Low |
| Wichmann, et al. (42), Germany, 2020 | Mixed-method study | Older adults (65-75 years). Questionnaire n=266 (120 males, 146 females), interviews n=25. | Questionnaire and semi-structured interviews after 10 weeks of using a web-based PA program, including self-monitoring and recommended exercises. | Facilitators:   - Easily performed exercises (no additional props needed) - Platform easily integrated into everyday life - Motivational activity tracker   Barriers:   - Content not individualized - Reasons to increase PA not sufficiently motivated - Insufficiently challenging or not age-appropriate exercises - PA^1^ diary time consuming - Technical issues with PA^1^ tracker (installation, synchronization, data saving)   Suggested improvements:   - Exercise instructions by video - Add goal setting - Add more varying exercises covering muscle strengthening, coordination and endurance - Provide platform instructions and technical support. | Medium |

^1^Phycical Activity (PA)
